# Classification of Acid and Alkaline Enzymes Based on Normalized Van der Waals Volume Features

**DOI:** 10.1002/prca.70009

**Published:** 2025-05-31

**Authors:** Hao Wan, Quan Zou, Yanan Zhang

**Affiliations:** ^1^ Institute of Advanced Cross‐Field Science College of Life Science Qingdao University Qingdao China; ^2^ Yangtze Delta Region Institute (Quzhou) University of Electronic Science and Technology of China Quzhou Zhejiang China

**Keywords:** 188D, acid enzyme, adaptation mechanisms, alkaline enzyme, key feature, machine learning

## Abstract

**Objective:**

Acidic and alkaline enzymes play crucial roles in the food industry and environmental management. This study aims to develop a computational method for accurately distinguishing between acidic and alkaline enzymes to enhance their stability in varying pH environments.

**Methods:**

We employed AutoProp for feature extraction and the MRMD3.0 algorithm for feature selection. The most discriminative feature, the normalized Van der Waals volume (nFeat43), was identified and used for classification.

**Results:**

The selected feature (nFeat43) achieved a classification accuracy of 76.2% in distinguishing acidic from alkaline enzymes. Further analysis was conducted to interpret the physicochemical significance of this feature in enzyme discrimination.

**Conclusions:**

Our findings demonstrate that nFeat43 is a key determinant in differentiating acidic and alkaline enzymes. This method provides a rapid and reliable computational approach for enzyme characterization, which could aid in industrial and environmental applications.

## 1 Introduction

Enzymes are highly effective biocatalysts and can be categorized as acidic or alkaline enzymes depending on the pH environment in which they work. Acidic and alkaline enzymes are essential in food, medicine, and environmental treatment and have high commercial value [1, 2, 3, 4, 5]. In the food industry, acidic enzymes are widely used in processes such as alcoholic fermentation, protein hydrolysis, and fur softening, while alkaline enzymes are essential components of biological detergents. Accurate identification and optimization of these enzymes can lead to more efficient and sustainable food processing methods. In medicine, understanding the pH stability of enzymes is crucial for designing stable therapeutic enzymes and drug delivery systems, particularly for treatments targeting acidic or alkaline environments within the human body. Furthermore, in environmental science, alkaline enzymes play a vital role in biodegradation and wastewater treatment, offering eco‐friendly solutions for pollution control. Most enzymes require the right environment to function. Between pH 6–8, most enzymes can work stably. However, enzyme activity decreases in some extreme environments (pH < 5 or pH > 9), limiting their application [6, 7, 8, 9]. Therefore, effectively identifying acidophilic and alkaliphilic bacteria and isolating their acidophilic and alkaliphilic enzymes has become a hot research topic. Meanwhile, by quantitatively comparing the differences in amino acid composition between acidophilic or basophilic enzymes and neutral enzymes, the mechanism of their stability in extreme pH environments can be explored, which can be helpful for drug design.

Although biochemical experiments can identify acidic enzymes and alkaline enzymes, this way is more time‐consuming and costly. Therefore, many scholars in the previous period used machine learning methods to discriminate acidic and alkaline enzymes efficiently [10, 11, 12, 13, 14, 15, 16]. They tried using different feature extraction methods and classification algorithms to improve accuracy. [17, 18] presented feature extraction based on protein secondary structure and a random forest classifier; the overall prediction accuracy was 90.4%. They suggested that enzyme stability in extreme pH environments is related to protein secondary structure. Using the same dataset, the work of [19] employed a fusion of multiple feature extraction methods to characterize protein sequences, including the use of Position‐Specific Scoring Matrix (PSSM) matrices [20, 21, 22] to reflect evolutionary information, average chemical shift, gene ontology, amino acid composition, reduced amino acid composition, the prediction accuracy was 94.01%. [23] removed the sequences with identity>25% from the original dataset to get the sequence containing 114 enzymes. Considering the hydrogen bonds in the secondary structure, [23] extended the dipeptide features to g‐gap dipeptide features, combined with the support vector machine [24, 25, 26, 27], to achieve 97.7% accuracy. Wang et al. [11] further divided the sequence into two parts and calculated the g‐gap dipeptide composition of the two parts separately, that is, dual g‐gap dipeptide composition. They modeled the prediction of acid and alkaline enzymes using a support vector machine, with a final accuracy of 95.9%. To reflect the positional information as well as the physicochemical properties among the residues, Khan et al. [10] used Split Amino Acid Composition (SAAC) and Pseudo Amino Acid Composition (PseAAC) feature extraction methods along with different classification algorithms, including probabilistic neural network (PNN), K‐nearest neighbor, decision tree, multi‐layer perceptron, and support vector machine, and achieved 99.2% accuracy. [12] summarized the progress of the digital features to express proteins and computational methods to identify acidic and alkaline enzymes.

Although the existing studies on the recognition of acidic and alkaline enzymes have a high accuracy of more than 90%, most studies are limited to improving the recognition efficiency of protein sequences. Few of them have been able to explore in depth the molecular mechanism of enzyme stability under acidic and alkaline conditions. To analyze the molecular mechanisms of enzyme molecules to maintain stability in acidic and alkaline environments, we reduced the feature dimensions in this study while maintaining high classification accuracy. We also kept a few essential features and looked into the relationship between feature meanings and amino acid structures. These findings provide a theoretical foundation for the molecular modification of enzyme proteins about their acidic and alkaline stability.

**FIGURE 1:**
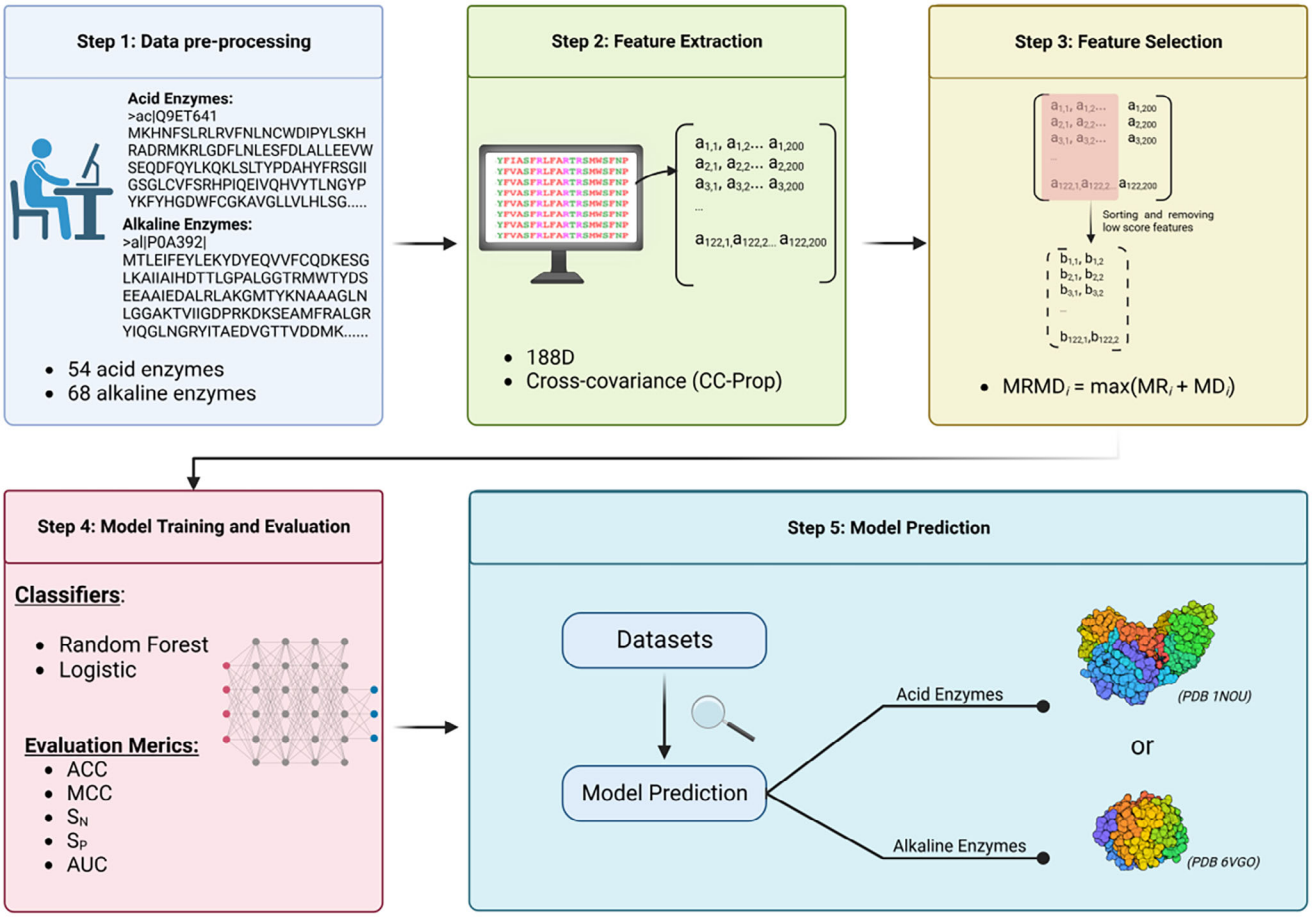
Diagram for constructing prediction models for alkaline and acid enzymes.

In this article, we first used AutoProps [28, 29] software to generate the feature matrices of acid and alkaline enzymes' collected amino acid sequences. Then we used MRMD3.0 [30, 31, 32] feature reduction software to remove the features with smaller contribution values, after which we further screened the retained features and uncovered the physicochemical properties of the amino acids reflected by the features to explain further the role of acid and alkaline enzyme in the structure and function of proteins, as shown in Figure 1. The protein structure and function differences between acidic and alkaline enzymes were further explained.

## 2 Methods

### 2.1 Data

Lin et al. [8] constructed the data used in this study, which contains sequences of 122 enzymes, including 54 acidic enzymes and 68 alkaline enzymes. All enzyme sequences were extracted from the BRENDA enzyme database [33]. Among them, the suitable pH of the acidic enzyme is below 5, and the proper pH of the alkaline enzyme is above 9. Pre‐processing of the sequences included the deletion of sequences less than 100 amino acids in length and the deletion of sequences with more than 25% similarity.

### 2.2 Feature Extraction

We transformed the features of the enzyme sequences into numerical matrices using the AutoProp feature extraction software [29, 34]. Using a built‐in classifier, the software returns the optimal features by comparing the results of 12 features, such as 188d, AAC, RAAC, and some combinations of features. The optimal features for this study are the combination of 188d and cross‐covariance features.

#### 2.2.1 188D

The 188D feature extraction method was proposed by Lin et al. [35]. It integrates the amino acid composition, the amino acids' eight most representative physicochemical properties, and the dipeptide distribution information in the protein sequence. The first 20 features of the feature vector (FV_1_–FV_20_) include the frequency of occurrence of each of the 20 amino acids (Equation 1).

(1) $$\;\left(FV_{1},FV_{2},\text{…},FV_{20}\right)=\left(\frac{n_{1}}{L},\frac{n_{2}}{L},\text{…},\frac{n_{20}}{L}\right)\;$$

where *n*_1_, *n*_2_, and *n*_20_ are the number of each of the 20 amino acids and *L* is the length of the sequence.

The following 168 features can be expressed based on the physicochemical properties of the amino acids. The 20 amino acids can be divided into three groups according to the numerical magnitude of each physicochemical property. Taking hydrophobicity (H) as an example, the 20 amino acids are categorized into three groups according to their hydrophobicity: RKEDQN, GASTPHY, and CVLIMFW, which are herein labeled as the “strong hydrophobic group,” the “moderately hydrophobic group,” and the “weakly hydrophobic group,” respectively. Three descriptors, content (C), distribution (D), and bivalent frequency (F), were used to characterize each protein, respectively. Through the three groups of amino acid contents (CH_1_, CH_2_, and CH_3_), the three content‐dependent feature vectors (FV_21_–FV_23_) can be defined by Equation (2).

(2) $$\;\left(FV_{21},FV_{22},FV_{23}\right)=\left(\frac{CH_{1}}{L},\frac{CH_{2}}{L},\frac{CH_{3}}{L}\right)\;$$

where *CH*_1_, *CH*_2_, and *CH*_3_ are the amino acid contents of the three groups, respectively. The subsequent 15 features are described by the distribution of amino acids in three groups at the 0%, 25%, 50%, 75%, and 100% sites of the sequence.

(3) $$\left\{\begin{array}{c} \;FV_{24},\text{…},FV_{28}=\left(\frac{DH_{11}}{L},\text{…},\frac{DH_{15}}{L}\right)\\ \;FV_{29},\text{…},FV_{33}=\left(\frac{DH_{21}}{L},\text{…},\frac{DH_{25}}{L}\right)\\ \;FV_{34},\text{…},FV_{38}=\left(\frac{DH_{31}}{L},\text{…},\frac{DH_{35}}{L}\right) \end{array}\right.$$

The subsequent three features count the bivalent frequency of two amino acids in different hydrophobicity groups.

(4) $$\;\left(FV_{39},FV_{40},FV_{41}\right)=\left(\frac{FH_{1}}{L-1},\frac{FH_{2}}{L-1},\frac{FH_{3}}{L-1}\right)\;$$

Therefore, 21‐dimensional features can be extracted from one physicochemical property so that 168‐dimensional features can be obtained from eight physicochemical properties.

#### 2.2.2 Cross‐Covariance (CC)

The CC variable measures the correlation between two residues along a sequence interval *lg* of two different properties [36, 37]. The formula is

(5) $$\mathrm{CC}\;\left(u_{1},u_{2},\mathrm{lg}\right)=\sum_{i=1}^{L-\mathrm{lg}}\left(P_{u_{1}}\left(R_{i}\right)-\bar{P_{u_{1}}}\right)\left(P_{u_{2}}\left(R_{i+\mathrm{lg}}\right)-\bar{P_{u_{2}}}\right)/\left(L-\mathrm{lg}\right)\;$$

where *i* represents the position of the amino acid in the sequence, *L* represents the length of the sequence, *lg* represents the distance between amino acids, *u*_1_ and *u*_2_ are different physicochemical indices of two different amino acids, *R_i_* and *R_i_*_+_ *_lg_*, *P_u_*_1_(*R_i_*) (*P_u_*_2_(*R_i_*_+_ *_lg_*)) is the value of the physicochemical index *u*_1_ (*u*_2_) for amino acid *R*_i_ (*R_i_*_+_ *_lg_*) at position *i* (*i* + *lg*). ${\bar{P}}_{u_{1}}\left({\bar{P}}_{u_{2}}\right)$ is the average of the entire sequence of physicochemical values of index *u*_1_ (*u*_2_). The dimension of the feature vector for CC feature extraction is N*(*N−*1)*LG, where *N* is the number of physicochemical properties, and *LG* is the maximum value of *lg* (*lg* = 1, 2,…, *LG*).

### 2.3 Feature Selection

Protein sequence information extracted from a single feature extraction method does not provide a comprehensive description of the various properties of proteins [38, 39], so the most common approach is to build a feature vector using multiple feature extraction methods to predict acid and alkaline enzymes. However, doing so poses some problems, such as generating many redundant features that reduce the accuracy of the predictive model. In addition, high‐dimensional features will inevitably increase the computational time and reduce the efficiency of prediction model operations [40, 41]. Feature selection can effectively remove redundant features and improve the computational efficiency of the model [42, 43, 44]. Feature selection methods, including the analysis of variance (ANOVA) [11, 23, 45, 46], principal component analysis (PCA) [47], minimal redundancy maximal relevance (mRMR) [48, 49, 50], increment of diversity (ID) [51], and maximum relevance maximum distance (MRMD) [30, 52–57] can be used in the prediction of acid and alkaline enzyme models to filter out the optimal features.

In this study, we utilized MRMD 3.0 to find the optimal subset of features. On the one hand, we relied on the feature selection algorithm to reduce the number of features and improve the model's accuracy. On the other hand, we relied on the feature selection algorithm to screen the critical protein physicochemical properties that differentiate acidic enzymes from alkaline enzymes. MRMD 3.0 belongs to the wraparound feature selection method, which is superior to the single‐filtered selection method. MRMD is the maximum relevance maximum distance, which utilizes the Pearson correlation coefficient to measure relevance and three distance functions to compute the redundancy. The more important the feature, the higher the correlation with the target vector, the larger the feature distance, and the lower the redundancy of the sub‐feature set, expressed as in Equation (6):

(6) $$\;MRMD_{i}=\max\left(MR_{i}+MD_{i}\right)$$

The maximum correlation coefficient *MR_i_* based on Pearson and the maximum distance *MD_i_* based on three distance matrices were calculated using the formula:

(7) $$\left\{\begin{array}{c} \max\;MR_{i}=\left|PCC\left(f_{i},\;C_{i}\right)\right|\left(1\leq i\leq M\right)\\ \max\;MD_{i}=ED_{i}+COS_{i}+TC_{i} \end{array}\right.$$

where PCC(·) denotes Pearson's correlation coefficient, *f_i_* represents the *i*‐th feature vector, *C_i_* denotes the class to which the sequence belongs, *M* denotes feature dimension, *ED_i_* denotes the Euclidean distance of the *i*‐th feature, *COS_i_* denotes the Cosine distance of the *i*‐th feature, and *TC_i_* denotes the Tanimoto coefficient of the *i*‐th feature.

### 2.4 Hyperparameter Tuning

For the Random Forest classifier, we used the default hyperparameters in Weka, including 100 trees and no limit on the maximum depth of the trees. These settings were chosen based on their ability to provide satisfactory performance without extensive tuning. In future work, we plan to explore the impact of hyperparameter tuning on model performance.

### 2.5 Performance Evaluation

The main evaluation indexes of the model, including sensitivity (*Sn*), specificity (*Sp*), accuracy (*ACC*), and Matthew's correlation coefficient (*MCC*), were calculated as follows [58, 59, 60, 61, 62, 63, 64]:

(8) $$Sn=\frac{TP}{TP+FN}$$

(9) $$Sp=\frac{TN}{FP+TN}$$

(10) $$ACC=\frac{TP+TN}{TP+FP+TN+FN}\;$$

(11) $$MCC=\frac{TP\times TN-FP\times FN}{\sqrt{\left(TP+FP\right)\left(TP+FN\right)\left(TN+FP\right)\left(TN+FN\right)}}$$

In addition, the ROC curve with false‐positive rate as the horizontal axis, true‐positive rate as the vertical axis, and the PR curve with recall rate as the horizontal axis and precision rate as the vertical axis were also used as evaluation indexes in this project [65, 66, 67, 68]. The area under the curve (AUC) is also used as a measure, and an AUC close to 1 indicates a better model prediction.

### 2.6 Computational Requirements

All computational experiments were conducted on a standard desktop computer with an Intel Core i7 processor and 16 GB of RAM. The feature extraction process using AutoProp and the feature selection process using MRMD3.0 were implemented in Python, with a total runtime of approximately 2 h for the entire dataset. Model training and evaluation were performed using the Random Forest classifier in Weka, with each 10‐fold cross‐validation run completing within 10 min. These modest computational requirements highlight the efficiency of our pipeline and its feasibility for broader applications.

## 3 Results and Discussion

### 3.1 Comparison of Classification Modeling Results

**FIGURE 2:**
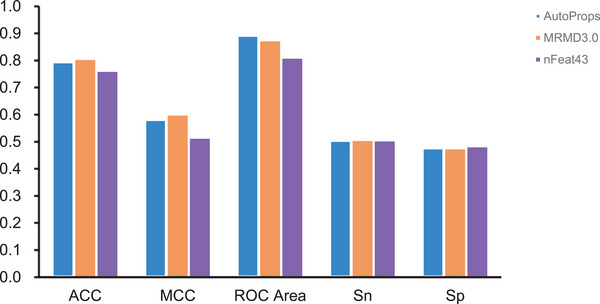
Performance comparison between classification models.

With AutoProps, 200 feature sets were extracted, and the classification model used a Random Forest classifier, which has an accuracy of 79.6%. After that, the number of features is reduced to 102 by using MRMD3.0 for dimensionality reduction, and the prediction model uses a random forest classifier, and the prediction accuracy rises to 80.6%. The performance metrics of every predictive model are shown in Figure 2. Also, from Table 1, we can observe that the value of MCC after dimensionality reduction using MRMD3.0 improves from 0.583 to 0.601, and the dimensionality reduced model performs better than the previous model in terms of accuracy and captures the complex relationships between the data better.

**TABLE 1:** Comparison of the performance outcomes of each classifier over various feature dimensions.

Methods	Feature number	ACC	MCC	ROC area	S_n_	S_p_	Classifier
AutoProps	200	0.796	0.583	0.894	50.60%	47.79%	RF
MRMD3.0	102	0.806	0.601	0.875	50.63%	47.58%	RF
nFeat43	1	0.762	0.515	0.811	50.53%	48.37%	Logistic

After further de‐redundancy, we found that only one feature can separate the acidic enzyme from the alkaline enzyme samples with a classification accuracy of 76.2% using a Logistic classifier. As shown in Table 1, while using nFeat43, the predictive performance of the classification model did not significantly degrade; instead, the specificity of the predictive model increased from 47.79% to 48.37%. This suggests that the nFeat43 feature can better capture the nuances of the data and effectively differentiate positive samples from negative ones.

### 3.2 Meaning of the Feature nFeat43

**FIGURE 3:**
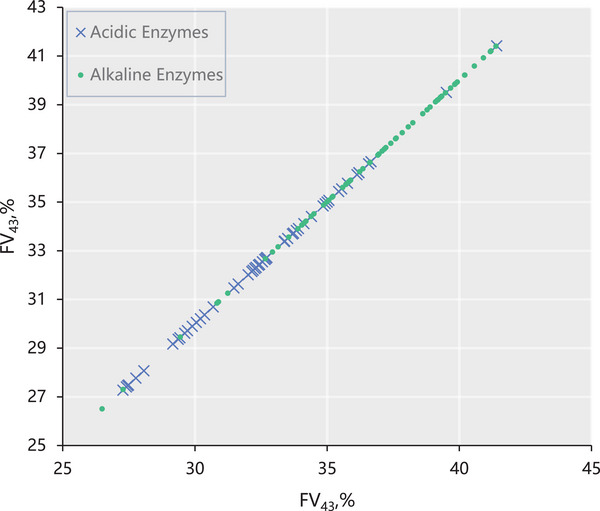
Scatter plot of nFeat43 feature value.

This is the 43rd‐ranked feature in the subset of features extracted from the 188‐dimensional physicochemical properties. According to the 188D feature extraction method mentioned in the METHODS section, the 43rd feature value indicates the content percentage of the medium group, Normalized van der Waals volume, which is the value after normalizing the van der Waals volume of each carbon atom in an amino acid. This value can help us understand the relative sizes of different amino acids in terms of their spatial structure to understand their physicochemical properties and biological functions. Alkaline enzymes have a higher probability of containing medium‐volume amino acids, and acidic enzymes have a lower likelihood of containing medium‐volume amino acids, as shown in Figure 3.

Acidic enzymes are mainly found in animal gastric juices, while alkaline enzymes are found primarily in microbial cells. Acidic enzymes are found in harsher environments and must be adapted to acidic environments. Hence, their structures need to be more stable, while medium‐sized amino acids tend to affect their structural stability. Studies have shown that enzyme stability increases linearly with van der Waals volume [69, 70]. There are also differences in substrate specificity between acidic and alkaline enzymes. Under certain conditions, acidic enzymes may be more likely to hydrolyze proteins into smaller peptide fragments, while alkaline enzymes may tend to produce larger peptide fragments. However, this trend may vary depending on the type of enzyme and the characteristics of the substrate. Therefore, acidic enzymes require a more robust hydrolysis capacity, while medium‐sized amino acids tend to affect their hydrolysis capacity. In summary, the possible reason that the medium‐volume amino acid composition of acidic enzymes is lower than that of alkaline enzymes is that acidic enzymes are exposed to a more demanding environment and require more robust structural stability and hydrolysis ability. In contrast, medium‐volume amino acids affect their structural stability and hydrolysis ability.

## 4 Conclusion

This study identified nFeat43, a normalized van der Waals volume feature, as a key factor for distinguishing between acidic and alkaline enzymes, achieving a classification accuracy of 76.2%. Our analysis revealed that alkaline enzymes contain more medium‐volume amino acids, while acidic enzymes have fewer, likely due to the need for greater structural stability in harsh acidic environments. This finding provides insights into enzyme adaptation mechanisms and offers a foundation for enzyme engineering and drug design.

## Author Contributions

Hao Wan performed data collection, analysis, and original draft of the manuscript. Yanan Zhang performed validation. Quan Zou performed proof‐reading. All authors have read and approved the final version of the manuscript for publication.

## Ethics Statement

The authors have nothing to report.

## Consent

The authors have nothing to report.

## Conflicts of Interest

The authors declare no competing interests.

## Data Availability

Publicly available datasets were analyzed in this study. The data can be retrieved from the following source: https://doi.org/10.1371/journal.pone.0075726.
